# Arithmetic learning in advanced age

**DOI:** 10.1371/journal.pone.0193529

**Published:** 2018-02-28

**Authors:** Laura Zamarian, Christoph Scherfler, Christian Kremser, Marie-Theres Pertl, Elke Gizewski, Thomas Benke, Margarete Delazer

**Affiliations:** 1 Department of Neurology, Medical University of Innsbruck, Innsbruck, Austria; 2 Neuroimaging Research Core Facility, Medical University of Innsbruck, Innsbruck, Austria; 3 Department of Radiology, Medical University of Innsbruck, Innsbruck, Austria; 4 Department of Neuroradiology, Medical University of Innsbruck, Innsbruck, Austria; Katholieke Universiteit Leuven, BELGIUM

## Abstract

Acquisition of numerical knowledge and understanding of numerical information are crucial for coping with the changing demands of our digital society. In this study, we assessed arithmetic learning in older and younger individuals in a training experiment including brain imaging. In particular, we assessed age-related effects of training intensity, prior arithmetic competence, and neuropsychological variables on the acquisition of new arithmetic knowledge and on the transfer to new, unknown problems. Effects were assessed immediately after training and after 3 months. Behavioural results showed higher training effects for younger individuals than for older individuals and significantly better performance after 90 problem repetitions than after 30 repetitions in both age groups. A correlation analysis indicated that older adults with lower memory and executive functions at baseline could profit more from intensive training. Similarly, training effects in the younger group were higher for those individuals who had lower arithmetic competence and executive functions prior to intervention. In younger adults, successful transfer was associated with higher executive functions. Memory and set-shifting emerged as significant predictors of training effects in the older group. For the younger group, prior arithmetic competence was a significant predictor of training effects, while cognitive flexibility was a predictor of transfer effects. After training, a subgroup of participants underwent an MRI assessment. A voxel-based morphometry analysis showed a significant interaction between training effects and grey matter volume of the right middle temporal gyrus extending to the angular gyrus for the younger group relative to the older group. The reverse contrast (older group vs. younger group) did not yield any significant results. These results suggest that improvements in arithmetic competence are supported by temporo-parietal areas in the right hemisphere in younger participants, while learning in older people might be more widespread. Overall, our study indicates that arithmetic learning depends on the training intensity as well as on person-related factors including individual age, arithmetic competence before training, memory, and executive functions. In conclusion, we suggest that major progress can be also achieved by older participants, but that interventions have to take into account individual variables in order to provide maximal benefit.

## Introduction

Older people, like younger people, need to acquire new fact knowledge and new skills to actively participate in modern society and adapt to its rapidly changing demands. Arithmetic knowledge is a core component of number cognition [1], and acquisition of arithmetic competence is a precondition for successful participation in modern society [2,3]. Indeed, individuals who have arithmetic difficulties face pronounced limitations in their autonomy in several personal and socio-economic aspects of every-day life (e.g., difficulty to read medication doses or bank invoices) [4]. Arithmetic knowledge can also be seen as an excellent model of declarative memory, common to most educated adults. Thus, arithmetic learning is a good instance for studying learning processes in advanced age. Healthy aging is characterised by structural and functional brain changes mostly affecting the prefrontal cortex [5,6]. Typically, older people show decreased memory, attention, and executive functions [6,7] which may affect performance on complex calculation and the acquisition of new arithmetic knowledge. This study aimed at elucidating the specific characteristics of arithmetic learning in advanced age.

Previous studies on number cognition have shown that some numerical abilities decline with age, while others remain stable over time [8]. A decrease in subitizing (simultaneous processing of a small number of visual items) [9] as well as a decrease in numerosity discrimination [10] (but see [11] for an alternative account) has been reported with increasing age. Other highly automated processes such as arithmetic fact retrieval (e.g., answering “3 x 3 = ?” or “5 + 2 = ?”) are found to be preserved in healthy older adults, although a decline in peripheral processing speed may slow down encoding and/or answering processes [12,13]. Age-related changes have been documented in complex computation or other numerical tasks that put high demands on executive functions such as flexibility and working memory [14,15]. With regard to procedural strategies, it has been shown that younger people and older people have a similar repertoire [8]. However, older adults use fewer strategies, adapt less flexibly their strategies to the problem characteristics, and are less efficient in the execution of procedural strategies than younger adults. These differences are in particular evident in difficult problems when the load on executive functions is increased [16–18]. Age differences have also been described in the volitional choice of strategies [19,20]. Older adults seem to avoid direct retrieval from memory and to rely strongly on back-up procedures [19,20]. This results in a delayed shift during intensive training from time-consuming computational strategies to memory-based strategies for the older adults relative to the younger adults [21,22]. Furthermore, older people show lower ability than younger people to process ratio concepts such as fractions, frequencies, and probabilities [23].

The acquisition of new arithmetic knowledge has been extensively studied in children and students [24–32]. A signature phenomenon of arithmetic skill acquisition is the shift from slow and effortful computations (e.g., “23 x 8 = (20 x 8) + (3 x 8) = 160 + 24 = 184”) to direct memory retrieval of problem-solution associations (“23 x 8 = 184”) [24,25,28,29]. Typically, developmental studies have investigated the acquisition of arithmetic fact knowledge (e.g., “3 x 5 = 15”), whereas experimental studies with adults have adopted more complex paradigms (e.g., alphabet arithmetic). Findings from these studies suggest that the learning rate depends on the number of item presentations, and that extended practice is necessary to develop automaticity in memory retrieval [25,26,29,33]. Following Rickard [29], at least 60 repetitions of an item are needed for a complete transition from computation-based strategies to fast and relatively effortless memory-based strategies. It has been also shown that practice reduces the demands on executive functions [27,34,35]. Studies with younger adults adopting functional magnetic resonance imaging (fMRI) methods have shown that a short but intensive training on complex arithmetic problems yields significant performance improvements (i.e., higher accuracy and faster response times) that go along with specific changes in brain activation patterns. Specifically, arithmetic skill acquisition following intensive training is associated with an activation decrease in fronto-parietal brain areas including the intraparietal sulcus (IPS) related to a reduced reliance on working memory and quantity processing, whereas a relative activation increase is found in parietal brain areas including the angular gyrus (AG) in association with an increased reliance on automated arithmetic fact retrieval [36–44] (but see [45] for a different point of view). It has also been shown that successful transfer of the newly acquired arithmetic knowledge (multiplication) to a new situation (related division) appears in association with activation in the left AG and is strongly influenced by inter-individual differences in arithmetic competence [41] (see also [38]). Successful transfer is a key feature of arithmetic competence. Even rehabilitation attempts cannot be considered complete and effective if a patient learns to retrieve facts from memory but is unable to adapt this knowledge to new problems [46].

Both behavioural and neuroimaging studies on arithmetic learning have mostly been carried out on younger adults. Few behavioural studies were done with older adults (e.g., [21,22,47]). It has yet to be explored in more depth whether arithmetic learning in older adults follows the same pattern as in younger adults. Furthermore, it is not known, for both younger adults and older adults, whether training-related improvements in arithmetic performance are associated with grey matter volume in specific brain areas. In this study, we investigated arithmetic learning in healthy older adults and compared their learning signature to learning in younger adults. In particular, we aimed at clarifying:

1. whether the number of problem repetitions is essential for the acquisition of new arithmetic facts in advanced age as it is for younger individuals [29];
2. whether older people are able to transfer the newly acquired knowledge (multiplication) to an unknown situation (related division) as younger people do [41];
3. whether training effects are larger for older people with lower prior arithmetic competence (for a study on younger adults see [38]);
4. whether training effects are long lasting in both age groups; and
5. which neuropsychological variables are good predictors of training-related improvements in arithmetic performance. This question is essential for older individuals as episodic memory and executive functions are known to decline with increasing age [6,7].
6. Finally, we investigated whether training-related performance improvements are associated with grey matter volume in specific brain areas. This question concerns both, younger and older individuals.

To these aims, participants (older adults, younger adults) were comprehensively tested before training on different neuropsychological measures (including memory and executive functions) and on experimental tasks of complex multiplication and division (e.g., “28 x 3 = ?”; “84: 3 = ?”). They then trained on two sets of complex multiplication problems for five consecutive days. This training procedure has proven to be successful for acquiring new arithmetic competence [42,43]. In this study, participants were presented with two sets of complex multiplication problems during training: one set having a high frequency of repetition (HF condition: 90 repetitions), the other set having a low frequency of repetition (LF condition: 30 repetitions). Participants were then tested on both trained (multiplication) and untrained (multiplication, division) problems immediately after training and after a 3-months break. After training, a subgroup of participants also underwent a morphometric MRI analysis.

We hypothesised that younger individuals would show faster learning of new arithmetic facts than older adults, as executive functions, learning, and episodic memory are known to decline with advanced age. We expected for both groups higher training-related performance improvements in the HF condition than in the LF condition. However, as cognitive decline in advanced age also affects executive resources, group differences might be stronger in the LF condition with less automated memory retrieval and higher demands on executive functions than in the HF condition. Moreover, as good executive functions are crucial for applying newly learned knowledge (multiplication) to a new situation (related division), older people might also show smaller transfer effects than younger people. At an individual level, we hypothesised that good memory functions and good executive functions are a precondition for efficient arithmetic learning since complex problems require the coordination of multiple steps, the maintenance of information in working memory, and the integration of partial results. We also hypothesized that training effects should be evident after a period of three months in both age groups. Stable effects of training over time are an index of profound modifications and are highly desirable for cognitive intervention and rehabilitation. Also, we expected to find larger performance improvements for people with lower prior arithmetic competence. Finally, we assumed to find a correlation between arithmetic performance and grey matter volume in parietal areas. Results of this study will be revealing about arithmetic skill acquisition in advanced age. They will also widen our understanding of older people’s specific training needs and give us new hints for the development of successful training methods for different age groups.

## Material and methods

### Estimation of sample size

Twenty young subjects participated in a pilot study on complex multiplication learning (72 repetitions, 10 problems). Based on accuracy scores at baseline, we classified 11 participants as “average” and 9 participants as “below average”. At post-training, the “below average” participants obtained a mean difference score in accuracy between trained and untrained problems of 41.11%. The “average” participants achieved a mean difference score of 23.18%. Based on these findings, we performed a sample-size estimation analysis. Results showed that at least 16 participants have to be included in each group to find a between-group difference of 17.93 (μ1 = 41.11, μ2 = 23.18, σ = 17.54; α = 0.05, two-sided; power 0.80, http://www.stat.ubc.ca/~rollin/stats/ssize/n2.html). Our previous fMRI studies on arithmetic learning tested about 18 participants. For this study, we planned to recruit at least 25 subjects in each age group in order to detect even smaller group differences as the one found in the pilot study.

### Participants

Twenty-five younger adults and 25 older adults participated in the study. The younger group had a mean age of 25.04 years (*SD* = 4.20, range 18–32); the older group had a mean age of 61.80 years (*SD* = 4.58, range 54–70). Groups did not differ from each other with regard to education (younger group: *M* = 14.04 years, *SD* = 2.61, range 10–18; older group: *M* = 14.08 years, *SD* = 3.08, range 10–18), one-way ANOVA, *p* >.1. They also had a comparable gender distribution (male:female in the younger group = 14:11; male:female in the older group = 13:12), *χ*^*2*^-test, *p* >.1. Please note that we did not find any significant differences between male participants and female participants in training and transfer effects (accuracy, response times). Older adults had a mean Mini Mental State Examination score of 29.32 (*SD* = 0.90, range 27–30) [48]. Participants were recruited by advertisement. None of them had a history of neurological or psychiatric disorders as determined by a screening interview. A total of 22 participants (13 younger adults, 9 older adults) agreed to undergo a neuroradiological investigation. Unfortunately, for technical reasons, the scanner was not available to assess all participants. An expert neurologist (C.S.) controlled the brain images and verified that they had no evidence of white matter lesions of grade 2 and 3, vascular or space-occupying lesions within the cerebrum, or motion artefacts [49]. This study was approved by the ethics committee of the Medical University of Innsbruck, Austria, and written informed consent was obtained from all individuals prior to participation. The study was carried out in accordance with the Declaration of Helsinki for experiments involving humans.

### Neuropsychological background tests

In the pre-training session (T1) and in the 3-months follow-up session (T3), participants performed a comprehensive neuropsychological background assessment including tests of verbal memory, verbal fluency, figural fluency, response inhibition, psychomotor speed, cognitive flexibility, verbal attention span, verbal working memory, and arithmetic processing (for details, see S1 Results).

### Assessment and training of multiplication

#### Materials

We selected 18 multiplication problems of comparable difficulty according to results of a pilot study performed with 10 volunteers. Problems were randomly assigned to three experimental conditions: untrained condition (n = 6), to-be-trained condition with high frequency of repetition (HF; n = 6), and to-be-trained condition with low frequency of repetition (LF; n = 6). This set size (n = 6) is standard in studies on arithmetic automaticity [26]. Multiplication problems were two-digit (range 12–28) times one-digit (range 3–8) problems. Solutions were always 2-digit numbers (range 45–98). Operands and solutions did not include numbers divisible by 10. All problems required a carrying procedure (e.g., “28 x 3 = 84”).

#### Pre-training, post-training, and 3-months follow-up assessments

We used SuperLab pro 2.0 for programming and running the training as well as the pre-training, post-training, and follow-up arithmetic assessments. Multiplication tasks were performed before training (*pre-training session*, *T1*), immediately after training (*post-training session*, *T2*), and 3 months after the last training session (*3-months follow-up session*, *T3*). The assessment of multiplication competence consisted of two blocks of 18 multiplication problems each. That is, the same 18 multiplication problems (n = 12 (to-be-)trained, n = 6 untrained) were administered twice at T1, T2, and T3. Order of problem presentation within a block was randomised. Participants were instructed to answer as accurately and as fast as possible. Both accuracy and reaction times with millisecond precision (RTs, msec) were recorded. Problems were presented at the centre of the computer screen as white characters on black background. Participants entered the problem solution on the number keypad of the computer keyboard. RTs were measured from the beginning of the problem presentation to the moment the participants typed in the first digit of the solution. Problems remained visible until the participant finished typing in the solution or until the time limit was reached (10 sec for the first digit, 1.5 sec for the second digit). Participants received a feedback only in case of timeout. The solution was not displayed to the participant.

#### Training

Individuals participated in five training sessions on five consecutive days. Training lasted from approximately 30 min in the first session to approximately 20 min in the last session. Within a session, participants performed 18 blocks, each containing two problems of the LF condition and six problems of the HF condition. Problems of the LF condition were therefore repeated six times within a session (30 times over the five training sessions), whereas problems of the HF condition were repeated 18 times within a session (90 times over the five training sessions). Order of problem presentation within a block was randomised. Participants were instructed to answer as accurately and as fast as possible. Both accuracy and RTs (msec) were recorded (see above for details). Problems remained visible until the correct solution was entered or until the time limit was reached (7 sec for the first digit, 1.5 sec for the second digit). Digits that were correctly and timely entered were displayed on the computer screen next to the equal sign of the problem (e.g., “28 x 3 = _ _” → “28 x 3 = 84”). Errors were not displayed. The complete correct solution remained visible on the computer screen for 1.5 sec. Participants received feedback in case of errors or timeout. The problem was then repeated until the participant entered a correct and timely solution. A problem was only presented when participants indicated their readiness by key press, making it possible for them to pause between trials. Before the first training session started, participants could familiarise with the number keypad of the computer keyboard by means of a short practice task.

### Assessment of division

#### Materials

Division problems were derived from the above mentioned 18 multiplication problems. For example, a multiplication problem such as “28 x 3” (= 84) was associated with the division problem “84: 3” (= 28). Division problems could be either related to the trained multiplication problems (transfer condition) or unrelated (no-transfer condition). There were therefore three types of division problems: unrelated division problems (n = 6; these were complementary to the multiplication problems of the untrained condition), related division problems of the LF condition (n = 6; these were complementary to the multiplication problems of the LF condition), and related division problems of the HF condition (n = 6; these were complementary to the multiplication problems of the HF condition). All problems were two-digit (range 45–98) divided by one-digit (range 3–8) problems, and the solutions were 2-digit numbers (range 12–28).

#### Pre-training, post-training, and 3-months follow-up assessments

At T1, T2, and T3, multiplication and division problems were tested separately. The assessment of division competence consisted of two blocks of 18 problems each. Order of problem presentation within a block was randomised (for other details, see above description of pre- and post-training tasks with multiplication problems). Participants were never informed that division problems could be related to the trained multiplication problems.

### Image acquisition and processing

MRI measurements were performed on a 3 Tesla whole-body MR scanner (Magnetom Verio, Siemens Erlangen, Germany) using a twelve-channel head coil. All participants underwent the same MRI protocol, including whole-brain T1-weighted, T2-weighted, and diffusion tensor imaging sequences. MRI parameters for sagittal T1-weighted 3D magnetization prepared rapid gradient echo (MPRAGE) were: repetition time (TR) = 1900 ms; echo time (TE) = 2.52 ms; inversion time (TI) = 900 ms; slice thickness: 1.0 mm; matrix: 256 × 246; number of excitations: 1; flip angel = 9°; field of view: 250 mm × 240 mm; voxel size: 0.98 mm x 0.98 mm x 1 mm. The acquisition time for the MPRAGE sequence was 4 min, 21 sec; the total duration of the imaging protocol was ca. 15 min.

To test the correlation of arithmetic learning with grey matter volume, T1-weighted MRI acquisitions were subjected to statistical parametric mapping (SPM, Wellcome Department of Cognitive Neurology, London, UK), a technique that objectively localizes focal changes of voxel values throughout the entire brain volume [50]. The software package SPM12 implemented in Matlab 7.8 (Mathsworks Inc., Sherborn, MA) was used to pre-process and analyse MRI data. VBM of grey and white matter compartments was achieved by using the standard version of the diffeomorphic anatomical registration using exponentiated lie algebra toolbox (DARTEL) implemented in SPM12 to have a high-dimensional normalization protocol [51]. Segmented and modulated images were transformed from group-specific diffeomorphic anatomical registration into Montreal Neurological Institute (MNI) space and smoothed by a Gaussian kernel of 8 x 8 x 8 mm. A masking threshold of 10% of the lower image signal was applied to reduce signal noise. For analysis of VBM, age and total intracranial volume were entered as covariates. MRI acquisitions were processed on a Dell Studio XPS 435 T workstation with 8 cores, each with a 2.93 GHz Intel 7 processor.

### Procedure

We explained participants that we were interested in investigating whether an intensive training on complex multiplication problems is effective and whether arithmetic skill acquisition is associated with other cognitive functions such as memory or ability to inhibit interference. We also told participants that, to investigate stability of training effects over time, a second comprehensive cognitive assessment was planned after about 3 months from the last training session. Exact details about assessments, study design, and hypotheses were not given. We also explained individuals to be interested in possible brain correlates of arithmetic competence and that they could participate in a neuroradiological investigation at post-training.

Table 1 gives a schematisation of the study procedure. At T1, participants performed a neuropsychological background assessment and the computer-based tasks assessing arithmetic competence with multiplication and division problems. Subsequently, participants performed at home five training sessions with multiplication problems on five consecutive days. The day after the last training session (T2), participants underwent a post-training assessment of arithmetic competence (multiplication, division). On the same day, a subgroup of participants (n = 22) also underwent MRI scanning of the brain. Cognitive functioning and arithmetic competence with multiplication and division were also tested 3 months after the last training session (T3). The pre-training, post-training, and 3-months follow-up assessments were done individually in a quiet room to minimise distraction. Training compliance and results were recorded by the computer program and were checked by L.Z. subsequently.

**Table 1:** Schematisation of the study procedure.

	Pre-training testing	Training	Training	Training	Training	Training	Post-training testing	3-months follow-up testing
	T1	Session 1	Session 2	Session 3	Session 4	Session 5	T2	T3
**Neuropsychological assessment**	Memory, Executive functions, Arithmetic processing							Memory, Executive functions, Arithmetic processing
**Arithmetic competence**	Multiplication^a^^,^^b^^,^^c^ Division^d^^,^^e^^,^^f^						Multiplication^a^^,^^b^^,^^c^ Division^d^^,^^e^^,^^f^	Multiplication^a^^,^^b^^,^^c^ Division^d^^,^^e^^,^^f^
**Training**		Multiplication^b^^,^^c^	Multiplication^b^^,^^c^	Multiplication^b^^,^^c^	Multiplication^b^^,^^c^	Multiplication^b^^,^^c^		

^(a)^ = untrained condition;

^(b)^ = trained condition with low frequency of repetition;

^(c)^ = trained condition with high frequency of repetition;

^(d)^ = unrelated condition;

^(e)^ = division condition related to the trained condition with low frequency of repetition;

^(f)^ = division condition related to trained condition with high frequency of repetition.

### Statistical analysis

#### Behavioral data

Statistical analyses were carried out with IBM SPSS Statistics–Version 24.0 for Windows (SPSS Inc., Chicago, IL, USA). Arcsine-transformed mean proportions of correct answers (2*arcsine(√x)) [52] and ln-transformed mean RTs in correct trials (outliers were excluded) were used in the analyses of response accuracy and response speed, respectively. Outliers were RTs slower or faster than 2.5 SD from the individual mean in each condition. Although analyses were performed on arcsine-transformed mean proportions of correct answers and ln-transformed mean RTs in correct trials, tables and figures report untransformed data. A Pearson correlation analysis was performed between training indexes at T2 and the corresponding training index at T3, as well as between the training index and the transfer index at T2 related to the HF condition. Indexes are measures of training and transfer effects in response speed (see below for an exact description). This analysis was performed for the whole sample as well as for the two age groups separately. A Pearson correlation analysis was also performed for the two groups separately to examine the relation of training and transfer effects at T2 (accuracy, RTs) with non-numerical cognitive functions (verbal memory: learning, immediate recall, delayed recall; executive functions: category-shifting verbal fluency, interference inhibition, psychomotor speed, cognitive flexibility, verbal attention span, verbal working memory) and prior arithmetic competence (average performance on experimental multiplication tasks at T1). Significant variables were then entered into a stepwise regression analysis to investigate which ones could best predict training and transfer effects in the two groups. The alpha level was set at .05.

#### Imaging data

A flexible factorial model was set up to test the interaction of training effects in response accuracy and response speed x group (younger adults, older adults) on grey matter volume. With regard to accuracy, we defined training effects as difference between trained multiplication problems in the HF condition and untrained multiplication problems. To assess training effects in response speed, we computed a *training index* as mean RTs with untrained problems minus mean RTs with trained problems of the HF condition, divided by mean RTs with both problem types. We focussed our analysis on HF problems as training effects were larger for this condition. Inferences were made at p < .001 following family-wise error (FWE) correction at the cluster level (p < .05) for multiple comparisons across the entire brain volume. In areas where there was a significant grey matter volume x group interaction, grey matter volume values were extracted for linear regression analysis with SPSS 24.0 (Chicago, IL, USA).

## Results

### Neuropsychological background tests

Results of the neuropsychological background assessments carried out at T1 and at T3 are reported in detail in the supplementary material (S1 Results). One younger participant did not participate in the 3-months follow-up assessment. Median scores of both groups were in the average range or above cut-off of standardised norms. At both T1 and T3, groups significantly differed from each other in tests of verbal memory and executive functions, with the older group scoring lower than the younger group. At T1, we also found a significant group difference in measures of arithmetic processing such that the older group scored higher than the younger group in arithmetic fact knowledge (e.g., “5 + 2 = ?”, “7–5 = ?”, “8 x 9 = ?”, “12: 6 = ?”) and written complex calculation. The group differences in arithmetic tests were not significant at T3. We found no other significant group difference.

The comparison of performance between sessions indicated significant improvements in time-related tests of executive functions for both groups. For the older group, we also found a performance improvement in approximate complex calculation and a performance decline in written complex calculation. Other differences were not significant.

### Multiplication

#### Age differences in pre-training performance (T1)

We report a detailed description of results of the pre-training session in the supplementary material (S2 Results). In sum, untrained and (to-be-)trained problems were of comparable difficulty for both age groups.

#### Age differences in training progression

**Fig 1:**
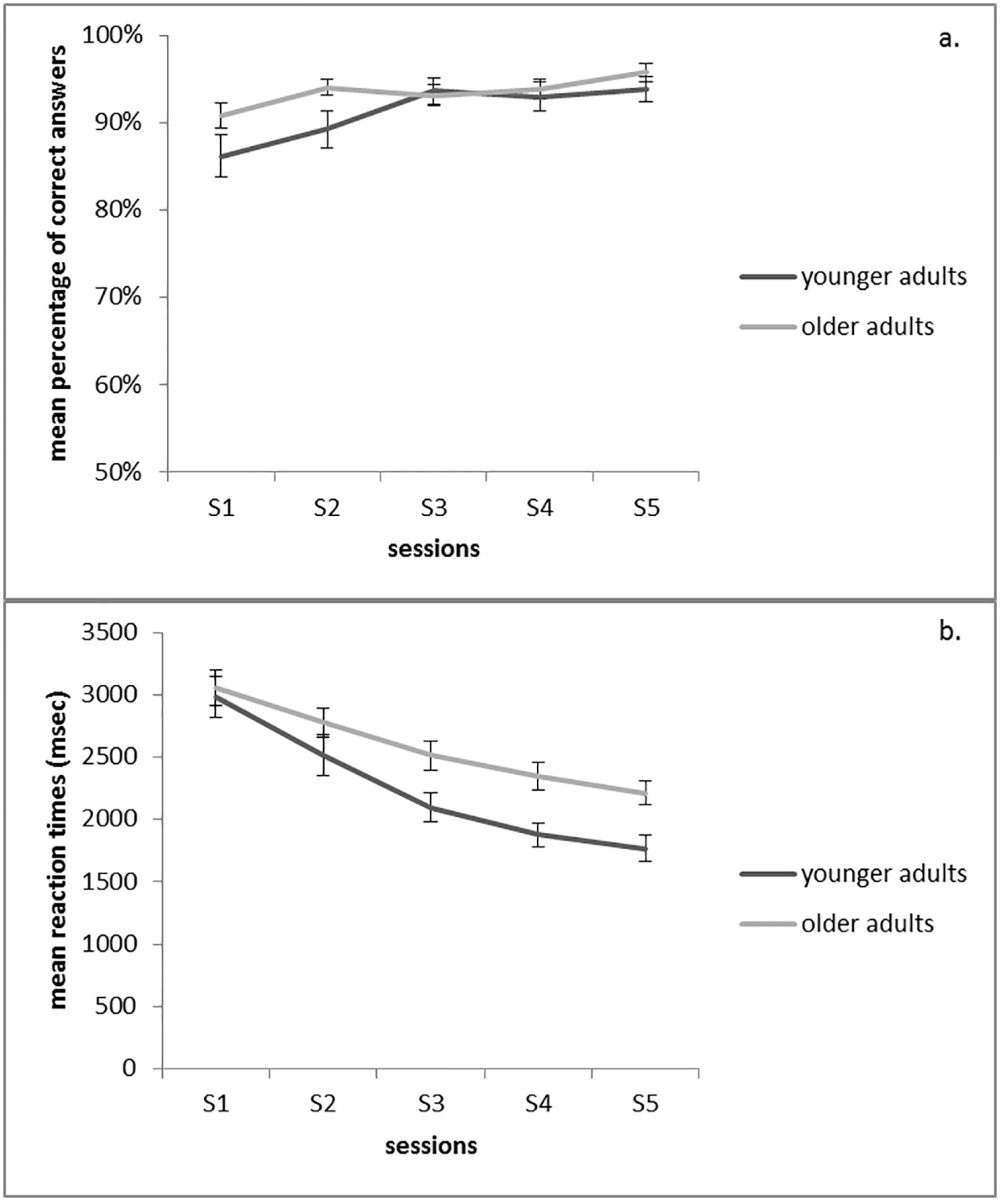
Mean percentage of correct answers (panel a) and mean reaction times in correct trials (panel b) as a function of training session (S1, S2, S3, S4, S5) and group (younger adults, older adults). Bars indicate the standard error of the mean.

A detailed description of results is reported in the supplementary material (S3 Results). In sum, both groups showed performance improvements during training (Fig 1). However, speed improvements were more pronounced for the younger group than for the older group. Both groups responded more accurately and faster to HF problems than to LF problems.

#### Age differences in post-training performance (T2)

As measure of training effects in response accuracy, we computed for each individual the difference in the arcsine-transformed mean proportion of correct answers between trained and untrained conditions. This measure was calculated for the HF and LF conditions, separately. As measure of training effects in response speed, we computed RT indexes. The *training index for the HF condition* was computed as mean ln-transformed RTs with untrained problems minus mean ln-transformed RTs with trained problems of the HF condition, divided by mean ln-transformed RTs with untrained problems. The *training index for the LF condition* was computed as mean ln-transformed RTs with untrained problems minus mean ln-transformed RTs with trained problems of the LF condition, divided by mean ln-transformed RTs with untrained problems. RT indexes have the advantage that they take individual differences in processing speed into account. Moreover, RT indexes control for possible delays in typing in the answer as typing time is included in the nominator (untrained RTs minus trained RTs) as well as in the denominator of the computation (untrained RTs [baseline performance]). Training effects in accuracy and in RTs were computed for T2 and T3, separately. In line with previous analyses, we report untransformed data. Larger positive values indicate larger training effects.

We carried out a mixed ANOVA on differences in the arcsine-transformed mean proportion of correct answers at T2 with condition (HF, LF) as within-subject factor and group (younger adults, older adults) as between-subjects factor. Results indicated a highly significant main effect of condition, *F*(1, 48) = 15.40, *MSE* = .79, *p* < .001, *μ*_*p*_^*2*^ = .24, with participants showing larger training effects with the HF condition than with the LF condition (HF: *M* = 18.33% correct, *SD* = 17.90; LF: *M* = 15.00% correct, *SD* = 18.52). The main effect of group failed to reach significance, *p* = .076. The interaction between condition and group was also not significant, *p* > .1, indicating a comparable performance between groups.

A mixed ANOVA, which was performed on training indexes at T2 with condition (HF, LF) as within-subject factor and group (younger adults, older adults) as between-subjects factor, indicated significant main effects of condition, *F*(1, 48) = 107.99, *MSE* = .02, *p* < .001, *μ*_*p*_^*2*^ = .69, and of group, *F*(1, 48) = 12.83, *MSE* = .02, *p* = .001, *μ*_*p*_^*2*^ = .21, but no significant interaction, *p* > .1. Younger participants obtained larger training indexes than older participants (younger group: *M* = .43, *SD* = .12; older group: *M* = .29, *SD* = .14). Also, training indexes were larger with the HF condition than with the LF condition (HF: *M* = .44, *SD* = .15; LF: *M* = .28, *SD* = .16).

In sum, at post-training (T2), training effects were larger with HF problems than with LF problems. While groups did not differ from each other with regard to accuracy, the younger group showed larger training effects in response speed than the older group.

#### Age differences in delayed post-training performance (T3)

A mixed ANOVA performed on differences in the arcsine-transformed mean proportion of correct answers at T3 with condition (HF, LF) as within-subject factor and group (younger adults, older adults) as between-subjects factor did not show any significant results, all *p* > .1.

We also performed a mixed ANOVA on training indexes at T3 with condition (HF, LF) as within-subject factor and group (younger adults, older adults) as between-subjects factor. Results indicated significant main effects of condition, *F*(1, 47) = 4.59, *MSE* = .04, *p* < .05, *μ*_*p*_^*2*^ = .09, and of group, *F*(1, 47) = 4.84, *MSE* = .00, *p* < .05, *μ*_*p*_^*2*^ = .09, but no significant interaction, *p* > .1. Training indexes were larger for the younger group than for the older group (younger group: *M* = .15, *SD* = .16; older group: *M* = .06, *SD* = .10), and with the HF condition than with the LF condition (HF: *M* = .12, *SD* = .17; LF: *M* = .08, *SD* = .15).

In sum, in the 3-months follow-up investigation (T3), younger adults showed larger training effects in RTs than older adults. Also, training effects in RTs were larger with HF problems than with LF problems. Results did not yield significance in the analysis of accuracy.

### Division

#### Age differences in pre-training performance (T1)

Results are described in detail in the supplementary material (S2 Results). In general, participants found the division problems related to the LF multiplication condition more difficult than the unrelated division problems and the division problems related to the HF multiplication condition. As this could make the interpretation of differences between conditions following training problematic, we decided to focus the following analyses on unrelated division problems and division problems related to the HF multiplication condition which did not differ from each other at T1.

#### Age differences in transfer effects

As measure of transfer effects in response accuracy, we computed for each individual the difference in the arcsine-transformed mean proportion of correct answers between related and unrelated conditions. As measure of transfer effects in response speed, we calculated a *transfer index* as follow: mean ln-transformed RTs with unrelated problems minus mean ln-transformed RTs with related problems, divided by mean ln-transformed RTs with unrelated problems. Transfer effects in accuracy and in RTs were computed for T2 and T3, separately. We report untransformed data. In general, larger positive values indicate larger transfer effects.

Age differences in transfer effects were analysed by means of a MANOVA with group (younger adults, older adults) as fixed factor. Transfer effects in accuracy and RTs computed for T2 and T3 separately were entered as dependent variables. Descriptive statistics are reported in Table 2. At T2, groups did not differ from each other with regard to transfer effects in response accuracy, whereas younger adults showed significantly larger transfer effects in RTs than older adults. At T3, transfer effects in response accuracy were significantly larger for the younger group than for the older group. The reverse pattern was found with regard to RTs, with the older group showing larger transfer effects than the younger group.

**Table 2:** Descriptive statistics of the analysis of transfer effects.

	Younger adults		Older adults		*F* value	*p*	*μ*_*p*_^*2*^
	Mean	SD	Mean	SD			
**Overall MANOVA**					F_4,43_ = 3.53	.014	.25
**Transfer effects in accuracy at T2 (% correct)**	8.00	20.76	3.33	23.69	F_1,46_ = 1.04	.314	.02
**Transfer effects in RTs at T2 (quotient)**	.14	.17	.04	.16	F_1,46_ = 5.47	.024	.11
**Transfer effects in accuracy at T3 (% correct)**	6.60	14.32	-5.00	14.23	F_1,46_ = 6.12	.017	.12
**Transfer effects in RTs at T3 (quotient)**	-.06	.31	.11	.14	F_1,46_ = 5.78	.020	.11

Transfer effects in response accuracy are differences between related division problems and unrelated problems. Transfer effects in response speed are mean RTs with unrelated problems minus mean RTs with related problems, divided by mean RTs with unrelated problems. Positive values indicate larger transfer effects.

To sum up the most important results with multiplication, we found that both groups significantly profited from training. The speed advantage with trained problems was, however, larger for the younger group than for the older group, not only immediately after training (T2) but also after a 3-months break (T3). The effect of problem repetition was comparable between groups at both T2 and T3, with participants showing larger performance improvements with HF problems than with LF problems. With regard to division, younger adults showed larger transfer effects in response speed at T2 than older adults. Group differences were less clear-cut at T3.

Please note that, with regard to training and transfer effects in RTs, we found the same results when we computed training and transfer indexes as in [41], where the differences between conditions were divided by the sum of RTs in both conditions.

### Relation between training and transfer (RT) indexes

A Pearson correlation analysis was carried out for the whole sample between training indexes at T2 and training indexes at T3, as well as between the training index and the transfer index at T2 related to the HF condition. We focus this analysis on RT indexes as training-related improvements in performance speed were particularly revealing. Results indicated that higher training effects at T2 were associated with higher training effects at T3 (HF: *r* = .366, *p* = .01; LF: *r* = .264, *p* = .067). Also, participants who profited more from multiplication training showed higher transfer effects to related division problems at T2 (*r* = .517, *p* < .001). When the analysis was carried out for the two age groups separately, the correlation between training and transfer indexes at T2 remained significant for the younger group only (*r* = .598, *p* < .01). Other results were not significant, all *p* > .05.

### Relation of training and transfer effects at T2 with cognitive functioning and prior arithmetic competence

We computed a Pearson correlation analysis for the two age groups separately between training and transfer effects at T2, neuropsychological measures, and prior arithmetic competence. Training and transfer effects in response accuracy and speed were defined as above (see analyses of post-training performance). Significant results are reported in Table 3. For the younger group, we found that lower prior arithmetic competence and lower executive functions (psychomotor speed, verbal working memory) correlated with higher training effects, and that higher executive functions (cognitive flexibility, verbal working memory) correlated with higher transfer effects. For the older group, lower memory and set-shifting correlated with higher training effects. Other correlations were not significant, all *p* > .05.

**Table 3:** Significant results of a Pearson correlation analysis for each age group separately.

	Training effects (T2)				Transfer effects (T2)	
	HF condition		LF condition		related to HF	
	accuracy	RTs	accuracy	RTs	accuracy	RTs
**Prior arithmetic competence (mean accuracy at T1)**	-.693**(Y)					
**Delayed recall (VMLT)**	-.453*(O)		-.419*(O)			
**Set-shifting (category-shifting verbal fluency, RWT)**			-.488*(O)			
**Psychomotor speed (TMT-A)**	.472*(Y)					
**Cognitive flexibility (TMT-B)**						-.610**(Y)
**Verbal working memory (digit span backward, WMS)**	-.411*(Y)					.400*(Y)

Y = significant correlation for the younger group; O = significant correlation for the older group. Training effects in response accuracy are differences between trained multiplication problems and untrained problems. Training effects in response speed are mean RTs with untrained problems minus mean RTs with trained problems, divided by mean RTs with untrained problems. Similarly, transfer effects in response accuracy are differences between related division problems and unrelated problems. Transfer effects in response speed are mean RTs with unrelated problems minus mean RTs with related problems, divided by mean RTs with unrelated problems. Positive values indicate larger training and transfer effects.

* p < .05.

** p < .01.

We also performed a series of regression analyses using the stepwise method to investigate which neuropsychological variables could predict training and transfer effects at T2. Results of the analysis performed for the younger group indicated “prior arithmetic competence” as a significant predictor of training effects in accuracy with HF problems (adjusted *R*^*2*^ = .457; *F*(1, 23) = 21.21, *p* < .001). “Cognitive flexibility” emerged as a significant predictor of transfer effects in RTs (adjusted *R*^*2*^ = .345; *F*(1, 23) = 13.64, *p* = .001). The analysis performed for the older group indicated “set-shifting” as a significant predictor of training effects in accuracy with LF problems (adjusted *R*^*2*^ = .205; *F*(1, 23) = 7.20, *p* < .05). We further found for the older group that “delayed recall” could explain 17.1% of variance in the training effects (accuracy) with HF problems (adjusted *R*^*2*^ = .171; *F*(1, 23) = 5.94, *p* < .05).

### Relation between training effects and grey matter volume

**Fig 2:**
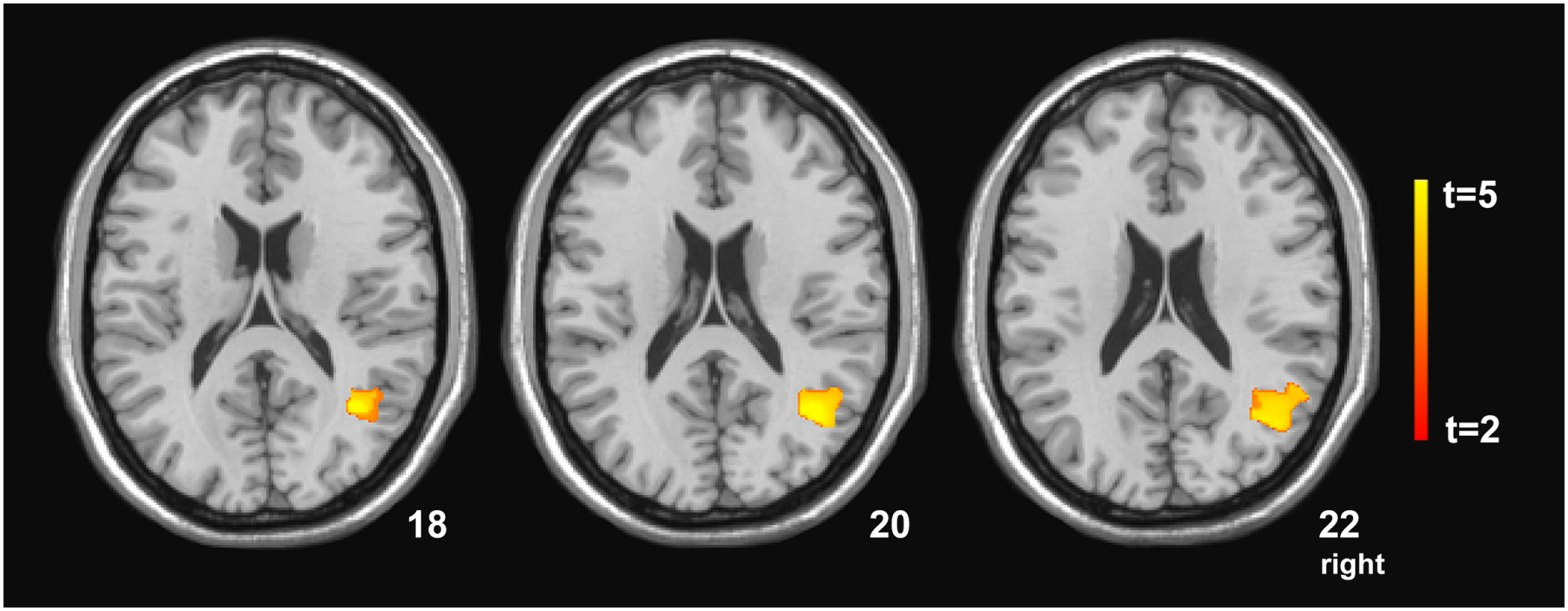
Statistical parametric mapping (*t*) intensity projection maps rendered onto a stereotactically normalized MRI scan, showing a voxel cluster of the significant interaction of increases of both grey matter volume and training effects in response accuracy for the younger group vs. the older group (statistical significance is thresholded at *p* < .001, FWE *p* < .05 corrected at the cluster level). The number at the bottom right corner of each MRI scan corresponds to the z coordinate in MNI space. The right side of the image corresponds to the right side of the brain.

Younger adults showed a significant positive correlation between training effects in response accuracy and grey matter volume of the bilateral postcentral gyri and the right inferior parietal lobule (*p* < .001, Table 4). No significant correlations were evident for older adults. A significant interaction between training effects in response accuracy and grey matter volume was evident for the younger group in contrast to the older group in the right middle temporal gyrus extending to the AG (*p* < .05; Table 4, Fig 2). The interaction effect was such that higher training effects in accuracy were related to increased grey matter volume only in the younger group (*r* = .801; *r*^*2*^ = .641; *p* < .001). The reverse contrast (older group vs. younger group) was not significant. No significant result was found in the analysis of RTs.

**Table 4:** Brain regions showing a positive correlation between training effects in response accuracy and brain volume for the younger group, and for the contrast younger group vs. older group.

Group / contrast	Brain regions	Cluster extent (voxels)	X	Y	Z	*t*-value	*p*-value corrected at cluster level	Height threshold
Positive correlation of grey matter volume with training effects in accuracy (*) for the younger group	Right inferior parietal lobule	1347	39	-57	54	7.88	0.002	0.001
	Right postcentral gyrus	1586	42	-11	48	6.48	0.001	0.001
	Left postcentral gyrus	1017	-59	-14	36	6.18	0.007	0.001
Interaction of grey matter volume and training effects in accuracy (*) in the younger group vs. the older group	Right middle temporal gyrus, extending to the angular gyrus	658	39	-60	18	5.44	0.038	0.001

XYZ coordinates reflect the peak *t*-value within each cluster reported in MNI space.

^(^*^)^ Training effects in response accuracy are defined as difference between trained problems (HF condition) and untrained problems.

We found no significant differences in training and transfer effects at T2 between subjects who participated in the neuroradiological investigation and subjects who did not, all *p* > .1.

## Discussion

This study aimed at elucidating the specific characteristics of arithmetic skill acquisition in advanced age. Here below, we summarise the main results with regard to the questions raised in the introduction.

### Age differences and stability of training and transfer effects over time

Results indicated that both groups (younger and older) significantly profited from intensive training of complex multiplication problems. After training, both younger adults and older adults responded faster and more accurately to trained multiplication problems than to untrained multiplication problems of comparable difficulty. Performance improvements were, however, larger for the younger participants than for the older participants. Although performance of both groups tended to decline over time, these group differences were significant not only immediately after training but also after a 3-months break. Younger adults could transfer better than older adults the newly acquired arithmetic knowledge (multiplication) to an unknown situation (related division) immediately after training.

### Effects of practice

In this study, we found that the number of problem repetitions during training had a significant effect. Both groups showed significantly better performance after 90 problem repetitions than after 30 problem repetitions. As suggested by previous studies [25,26,29,33], the frequency of item repetitions is a crucial factor in the acquisition of arithmetic fact knowledge. Older adults showed lower training effects than younger adults in both, LF and HF conditions. Recent brain imaging studies have shown that the repetition rate may also determine which brain structures are involved in arithmetic processing. While the studies by Delazer and colleagues [37–39,41] reported a relatively higher activation in parietal areas (AG) after intensive training with up to 90 repetitions of a small set of problems, Bloechle at al. [45] found the hippocampus to be critically involved after a lower number of problem repetitions with a larger set of problems. Thus, different brain structures may be implicated over time in the learning process and in the gradual acquisition of new fact knowledge. Hippocampal structures seem to be essential for the consolidation of arithmetic facts which may be retrieved from memory after sufficient repetitions.

### Effects of prior arithmetic competence

Regarding the influence of prior arithmetic performance, we found that training effects in response accuracy were stronger for younger participants with lower prior arithmetic competence than for younger participants with higher prior arithmetic competence. This result was not significant for the older adults. Prior arithmetic competence also emerged as significant predictor of training effects in accuracy of the younger group. One may hypothesize that people with high prior arithmetic competence already performed at their optimal level and thus did not show further improvements through training. Our study is in line with previous investigations reporting higher training and transfer effects for younger individuals with lower arithmetic competence [38,41]. For older adults, we found that training effects were related to non-numerical cognitive functions. This is further discussed below.

### Cognitive predictors of training and transfer effects

One further aim of this study was to assess the relation between individual variables–including memory and executive functions–and individual learning progress. Results of our correlation analysis indicated that different individual variables (prior arithmetic competence and executive functions for the younger group, memory and executive functions for the older group) play a relevant role in arithmetic learning and in the successful transfer of the newly learned arithmetic information. For older adults, we found that training effects were larger for those participants who scored lower at baseline in memory and executive-functions tests. Similarly, younger people with lower prior arithmetic competence and lower executive functions showed higher benefits from intensive arithmetic training. For the younger group, we also found that higher transfer effects were related to better executive functions. These findings were further explored by a regression analysis. For the older group, set-shifting emerged as a significant predictor of training effects in the LF condition, while delayed memory was a significant predictor of training effects in the HF condition. For the younger group, prior arithmetic competence emerged as a significant predictor of training effects in the HF condition, whereas cognitive flexibility was a significant predictor of transfer effects. All in all, these results confirmed and extended our expectations. The finding that low memory and executive functions predict high training effects in older adults seems counterintuitive at first glance. However, we may hypothesize that intensive training is associated with a general beneficial effect on several cognitive functions as indeed shown by [53]. Following this assumption, participants with low memory and executive functions might profit more from intensive training, while participants with high cognitive functions might already exploit their potential at the beginning. This hypothesis remains to be tested empirically. Altogether, these findings may be encouraging for intervention programmes: Especially those individuals with lower cognitive functions at baseline should benefit from targeted interventions.

### Arithmetic learning and the brain

This study also examined the relation between training-related performance improvements and grey matter volume in a subgroup of participants. A VBM analysis showed a significant positive correlation for younger participants between training effects in response accuracy and grey matter volume of the right inferior parietal lobule and the bilateral postcentral gyri. Older participants did not show any significant correlations. When the two groups were contrasted to each other, we found a significant interaction between higher training effects in response accuracy and increases of grey matter volume of the right middle temporal gyrus extending to the AG for the younger group relative to the older group. The reverse contrast (older group vs. younger group) did not yield any significant results. In sum, younger individuals with larger structural volume in the right parietal areas after training had learned more than those with smaller structural volume. There was no such correlation for the older adults. As grey matter volume and cortical thickness decrease with age [54], older adults relative to younger adults may show reduced associations between success in the acquisition of arithmetic competence and brain volume. This study together with very recent investigations using clinical, neuroimaging, and reversible inhibition methods [55] points to the contribution of the right hemisphere in calculation.

### Limitations

We should acknowledge two limitations of our MRI investigation. First, our sample of older adults undergoing MRI was quite small (9 older adults vs. 14 younger adults), and this might have hampered our success in detecting significant correlations between arithmetic improvements and brain volume. Second, our MRI investigation was cross-sectional, comparing the brains of different individuals who performed a similar arithmetic training but possibly had previous brain differences. In a pre- / post-training longitudinal study, we might be able to detect structural brain changes in the adult brain that are specifically related to arithmetic learning, deepening our understanding of experience-related brain plasticity and providing important insights for rehabilitation of brain damage patients.

A further limitation of this study was that we focused on cognitive performance variables and did not investigate metacognitive factors which may significantly influence the acquisition of new facts [19,20]. Older adults typically show a reluctance to use memory retrieval strategies instead of back-up strategies. Older adults seem to be less confident in memory retrieval, to put less weight on efficient processing, and to rely on slow computational strategies even when they have acquired memory representations. As shown by Touron and colleagues [21,22], this is reflected in a delayed shift during training from use of computational strategies to memory-based strategies in older adults relative to younger adults. In this investigation, we did not investigate age-related differences in metacognition. We do assume, however, that metacognition may be a relevant factor in the acquisition of new arithmetic knowledge and suggest that metacognition should be investigated in future research.

### Conclusions

Results of this study thus show that healthy older adults profit from intensive training. They are able to acquire new arithmetic knowledge and to retrieve this knowledge quickly and accurately from memory. Comparing the performance of older individuals with that of younger individuals, some differences appear. Although older adults similarly to younger adults show better performance with an increasing number of repetitions, they profit less than younger adults. Training programmes and rehabilitation approaches may thus take into account that older individuals are well able to acquire new numerical competence but may need a higher number of item repetitions in order to consolidate the new knowledge and to exploit their full learning potential. As regards training and rehabilitation, transfer of newly acquired skills to unknown situations is of major importance. In both younger and older groups, the majority of participants showed transfer of knowledge to new, untrained problems. However, transfer was better in younger people than in older people. As the capacity to successfully transfer is predicted in younger adults by cognitive flexibility, lower executive functions in advanced age could be a limiting factor which should be considered in rehabilitation approaches. Finally, our results show a significant decline of performance in a follow-up session indicating the necessity of constant training or booster sessions. Ideally, newly acquired skills (e.g., basic calculation skills) are used in everyday life and are thus maintained at a high level. As regards the brain imaging investigation, our findings add to previous studies. Arithmetic learning is associated with parietal brain structures in healthy younger individuals (in our findings, right lateralised), while learning in older individuals might recruit more distributed brain structures.

In conclusion, we see that the acquisition on new arithmetic knowledge is influenced by age and that major progress can also be achieved by older participants. In particular, interventions should take into account individual variables in order to provide maximal benefit. As shown by this study, people with lower arithmetic competence and lower cognitive functions prior to intervention may particularly profit from intensive training.

## Supporting information

S1 Results Neuropsychological background tests and scores obtained at T1 and T3 during the neuropsychological assessment. (DOCX)

S2 Results Accuracy and reaction times in computerised tasks assessing competence with multiplication and division problems at T1. (DOCX)

S3 Results Accuracy and reaction times with “trained” multiplication problems during the five sessions of training. (DOCX)

S1 Behavioural data (SAV)
