# Supplementary material for: Arithmetic learning in advanced age
Source: PLoS One. 2018 Feb 28;13(2):e0193529. doi: 10.1371/journal.pone.0193529 (PMC5831411; doi:10.1371/journal.pone.0193529)
Supplement: S1 Results — (DOCX) [file pone.0193529.s001.docx]

**S1 Supporting Information - Neuropsychological background tests and scores obtained at T1 and T3 during the neuropsychological assessment**

**Tests**

In the pre-training session (T1), participants performed a comprehensive neuropsychological background assessment including tests of verbal memory (VLMT) [1], verbal fluency (RWT) [2], figural fluency (5-points test) [3], response inhibition (FWIT) [4], psychomotor speed (TMT-A) [5], cognitive flexibility (TMT-B) [5], verbal attention span (digit span forward, WMS) [6], verbal working memory (digit span backward, WMS) [6], and arithmetic processing (NPC) [7]. Participants were also comprehensively assessed during the 3-months follow-up session (T3) to control for possible cognitive changes. At T3, the battery included parallel versions of the tests assessing verbal memory and verbal fluency, and the same tests we used in T1 to assess figural fluency, response inhibition, psychomotor speed, cognitive flexibility, verbal attention, verbal working memory, and arithmetic processing.

**Results**

Descriptive statistics are reported in Table S1 (pre-training) and Table S2 (3-months follow-up). One younger participant did not participate in the 3-months follow-up assessment. In both sessions, median scores of both groups were in the average range or above cut-off of standardised norms. Group comparisons were performed by means of Mann-Whitney U-tests. At T1 (Table S1), groups significantly differed from each other in tests of verbal memory, figural fluency, response inhibition, psychomotor speed, cognitive flexibility, arithmetic fact knowledge, and written complex calculation, all *p* < .05. Specifically, older adults scored lower than younger adults in tests of verbal memory and executive functions; younger adults scored lower than older adults in arithmetic facts and written calculation tests. Group performed comparably in tests of verbal fluency, verbal attention, verbal working memory, mental complex calculation, approximate complex calculation, and arithmetic principles, all *p* > .05.

At T3 (Table S2), groups significantly differed from each other in measures of verbal memory, figural fluency, response inhibition, psychomotor speed, and cognitive flexibility, all *p* < .05. In all measures of arithmetic competence and in tests of verbal fluency, verbal attention span, and verbal working memory, group differences were not significant, all *p* > .05.

We compared performance between sessions by means of Wilcoxon tests for those neuropsychological background tests that were administered at both T1 and T3 (comparisons between parallel versions of a test were not performed). Results indicated a significant performance improvement for the younger group in tests of figural fluency, response inhibition, psychomotor speed, and cognitive flexibility, all *p* < .05. For the older group, we found a significant performance improvement in tests of response inhibition, psychomotor speed, and approximate complex calculation, and a significant performance decline in written complex calculation, all *p* < .05. In other tests, performance of the two groups was comparable between sessions, all *p* > .05.

**Table S1. Medians, Interquartile Ranges, and Effect Sizes on Neuropsychological Background Measures at T1 for the Younger Group and the Older Group.**

|  |  | **Younger adults** | | **Older adults** | |  |
| --- | --- | --- | --- | --- | --- | --- |
|  | **Max. Score** | **Mdn** | **IQR** | **Mdn** | **IQR** | **Cohen’s *d*** |
| **Verbal memory (VLMT-A)** |  |  |  |  |  |  |
| **Learning (total)** | 75 | 62.00 | 57.00-65.00 | 54.00 | 47.00-62.00 | 0.86* |
| **Immediate recall** | 15 | 14.00 | 12.00-14.00 | 12.00 | 9.00-14.00 | 0.57 |
| **Delayed recall** | 15 | 13.00 | 12.00-15.00 | 13.00 | 9.00-14.00 | 0.60 |
| **Recognition (correct hits minus false positive)** | 15 | 15.00 | 14.00-15.00 | 15.00 | 13.00-15.00 | 0.44 |
| **Executive functions** |  |  |  |  |  |  |
| **Verbal fluency (RWT)** |  |  |  |  |  |  |
| **Semantic verbal fluency (animals/min)** |  | 28.00 | 27.00-31.00 | 28.00 | 25.00-31.00 | 0.25 |
| **Phonemic verbal fluency (s-words/min)** |  | 15.00 | 13.00-19.00 | 17.00 | 14.00-19.00 | -0.21 |
| **Category-shifting (sports-fruits/min)** |  | 16.00 | 16.00-18.00 | 16.00 | 14.00-17.00 | 0.59 |
| **Figural fluency (5-points test) (correct minus errors)** |  | 41.00 | 38.00-48.00 | 34.00 | 27.00-37.00 | 1.13*** |
| **Interference inhibition (FWIT) (sec)** |  | 69.00 | 58.00-83.00 | 81.00 | 74.00-93.00 | -0.77** |
| **Psychomotor speed (TMT-A) (sec)** |  | 20.00 | 18.00-24.00 | 29.00 | 27.00-36.00 | -1.07*** |
| **Cognitive flexibility (TMT-B) (sec)** |  | 46.00 | 39.00-60.00 | 61.00 | 54.00-77.00 | -0.76** |
| **Verbal attention span (digit span forward, WMS)** | 8 | 7.00 | 6.00-7.00 | 6.00 | 6.00-8.00 | 0.29 |
| **Verbal working memory (digit span backward, WMS)** | 7 | 5.00 | 4.00-6.00 | 5.00 | 4.00-5.00 | 0.11 |
| **Arithmetic processing (NPC)** |  |  |  |  |  |  |
| **Arithmetic facts (oral answer)** | 116 | 113.00 | 110.00-115.00 | 115.00 | 113.00-116.00 | -0.66* |
| **Mental complex calculation (oral answer)** | 20 | 18.00 | 15.00-19.00 | 18.00 | 18.00-19.00 | -0.52 |
| **Written complex calculation (written answer)** | 9 | 8.00 | 8.00-8.00 | 9.00 | 9.00-9.00 | -1.09*** |
| **Approximate complex calculation (multiple-choice)** | 12 | 10.00 | 9.00-11.00 | 11.00 | 10.00-11.00 | -0.20 |
| **Arithmetic principles (oral answer)** | 30 | 29.00 | 28.00-30.00 | 29.00 | 28.00-29.00 | 0.05 |

*Note.* Group comparisons were performed by means of Mann-Whitney *U*-tests. Mdn = median; IQR = interquartile range; VLMT-A = Verbal Learning Memory Test; RWT = Regensburger Wortflüssigkeitstest; FWIT = Farb-Wort Interferenz Test; TMT = Trail Making Test; WMS = Wechsler Memory Scale; NPC = Number Processing and Calculation. * *p* < .05. ** *p* < .01. *** p < .001.

**Table S2. Medians, Interquartile Ranges, and Effect Sizes on Neuropsychological Background Measures at T3 for the Younger Group and the Older Group.**

|  |  | **Younger adults** | | **Older adults** | |  |
| --- | --- | --- | --- | --- | --- | --- |
|  | **Max. Score** | **Mdn** | **IQR** | **Mdn** | **IQR** | **Cohen’s *d*** |
| **Verbal memory (VLMT-C)** |  |  |  |  |  |  |
| **Learning (total)** | 75 | 63.50 | 59.00-67.00 | 60.00 | 52.00-64.00 | 0.49 |
| **Immediate recall** | 15 | 14.50 | 12.00-15.00 | 12.00 | 10.00-15.00 | 0.46 |
| **Delayed recall** | 15 | 14.50 | 12.00-15.00 | 13.00 | 11.00-14.00 | 0.50* |
| **Recognition (correct hits minus false positive)** | 15 | 15.00 | 14.00-15.00 | 14.00 | 13.00-15.00 | 0.51 |
| **Executive functions** |  |  |  |  |  |  |
| **Verbal fluency (RWT)** |  |  |  |  |  |  |
| **Semantic verbal fluency (food/min)** |  | 25.50 | 23.50-31.50 | 25.00 | 24.00-29.00 | 0.29 |
| **Phonemic verbal fluency (p-words/min)** |  | 11.50 | 9.50-16.00 | 10.00 | 9.00-14.00 | 0.36 |
| **Category-shifting (cloths-flowers/min)** |  | 16.00 | 13.00-18.00 | 16.00 | 14.00-19.00 | -0.23 |
| **Figural fluency (5-points test) (correct minus errors)** |  | 47.50^a^ | 40.50-51.50 | 35.00 | 32.00-40.00 | 1.29*** |
| **Response inhibition (FWIT) (sec)** |  | 60.50^a^ | 54.50-73.50 | 79.00^a^ | 72.00-88.00 | -0.98** |
| **Psychomotor speed (TMT-A) (sec)** |  | 17.00^a^ | 14.50-20.00 | 25.00^a^ | 22.00-31.00 | -1.19*** |
| **Cognitive flexibility (TMT-B) (sec)** |  | 40.00^a^ | 35.50-51.00 | 58.00 | 51.00-77.00 | -1.33*** |
| **Verbal attention span (digit span forward, WMS)** | 8 | 7.00 | 6.50-8.00 | 7.00 | 6.00-8.00 | 0.23 |
| **Verbal working memory (digit span backward, WMS)** | 7 | 5.50 | 4.00-6.50 | 5.00 | 4.00-6.00 | 0.24 |
| **Arithmetic processing (NPC)** |  |  |  |  |  |  |
| **Arithmetic facts (oral answer)** | 116 | 114.00 | 112.50-116.00 | 115.00 | 113.00-116.00 | -0.29 |
| **Mental complex calculation (oral answer)** | 20 | 19.00 | 17.00-19.00 | 19.00 | 17.00-20.00 | -0.18 |
| **Written complex calculation (written answer)** | 9 | 8.00 | 8.00-9.00 | 8.00^b^ | 8.00-9.00 | -0.37 |
| **Approximate complex calculation (multiple-choice)** | 12 | 10.00 | 9.50-12.00 | 11.00^a^ | 11.00-12.00 | -0.34 |
| **Arithmetic principles (oral answer)** | 30 | 28.00 | 27.00-30.00 | 30.00 | 27.00-30.00 | -0.15 |

*Note.* Group comparisons were performed by means of Mann-Whitney *U*-tests. Mdn = median; IQR = interquartile range; VLMT-A = Verbal Learning Memory Test; RWT = Regensburger Wortflüssigkeitstest; FWIT = Farb-Wort Interferenz Test; TMT = Trail Making Test; WMS = Wechsler Memory Scale; NPC = Number Processing and Calculation. (a) = significant performance increase at T3 relative to T1 as indicated by Wilcoxon tests; (b) = significant performance decrease at T3 relative to T1 as indicated by Wilcoxon tests. * *p* < .05. ** *p* < .01. *** p < .001.

**Reference list**

[1] Helmstaedter C, Lendt M, Lux S. Verbaler Lern- und Merkfähigkeitstest. Göttingen: Belz Test GmbH; 2001.

[2] Aschenbrennen S, Tucha O, Lange KW. Regensburger Wortflüssigkeits-Test. Göttingen: Hogrefe; 2001.

[3] Haid TH, Martl C, Schubert F, Wenzl M, Kofler M, Saltuari L. Hamasch 5 Point Test-Revised (H5PT-R)—Additional normative data. 2004.

[4] Bäumler G. Farb-Wort-Interferenztest (FWIT) nach J.R. Stroop. Göttingen: Hogrefe; 1985.

[5] Lezak MD. Neuropsychological assessment. Oxford University Press; 1985.

[6] Härting C, Markowitsch HJ, Neufeld H, Calabrese P, Deisinger K. WMS-R Wechsler Gedächtnistest—Revidierte Fassung. Bern: Hans Huber; 2000.

[7] Delazer M, Girelli L, Granà A, Domahs F. Number processing and calculation--normative data from healthy adults. Clin Neuropsychol 2003;17:331–50. doi:10.1076/clin.17.3.331.18092.
