# Supplementary material for: Arithmetic learning in advanced age
Source: PLoS One. 2018 Feb 28;13(2):e0193529. doi: 10.1371/journal.pone.0193529 (PMC5831411; doi:10.1371/journal.pone.0193529)
Supplement: S2 Results — (DOCX) [file pone.0193529.s002.docx]

**S2 Supporting Information – Accuracy and reaction times in computerised tasks assessing competence with multiplication and division problems at T1**

**Multiplication**

**Results**

A mixed ANOVA with condition (untrained problems, (to-be-)trained problems of the LF condition, (to-be-)trained problems of the HF condition) as within-subject factor and group (younger adults, older adults) as between-subjects factor was performed on the arcsine-transformed mean proportion of accurate answers and on the ln-transformed mean RTs in correct trials, separately. Results of both analyses were not significant (Fig S1), all *p* > .1, indicating that the three conditions were of comparable difficulty for both groups at T1.

**Fig S1. Mean percentage of correct answers (panel a) and mean reaction times in correct trials (panel b) as a function of condition (untrained problems, (to-be-)trained problems of the LF condition, (to-be-)trained problems of the HF condition). Bars indicate the standard error of the mean.**


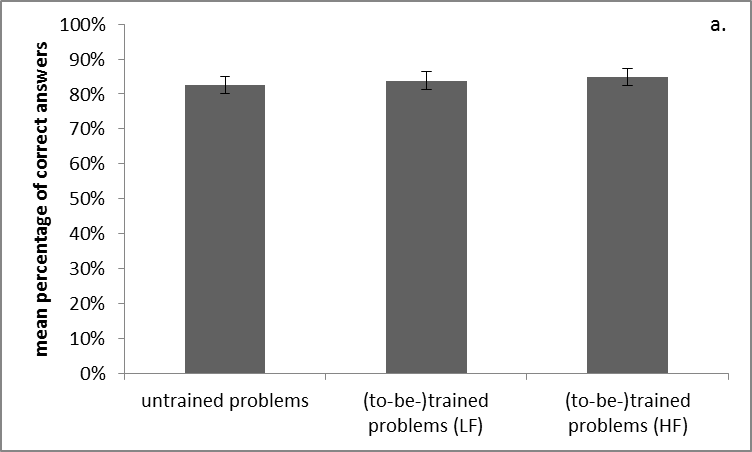

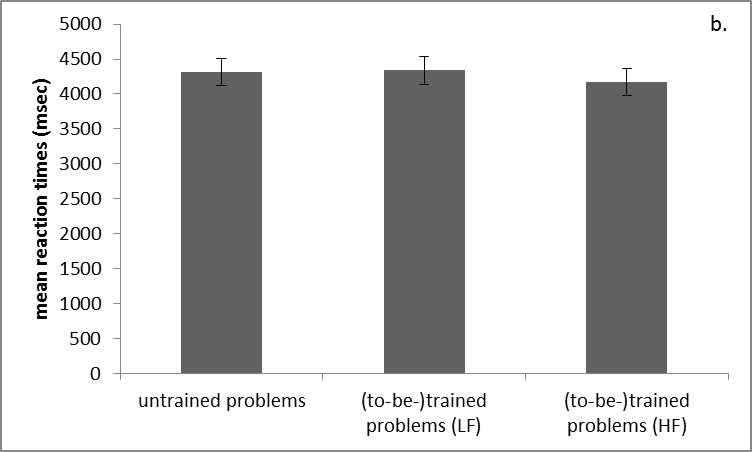


**Division**

**Results**

**Accuracy.** A mixed ANOVA with condition (unrelated division problems, division problems related to multiplication problems of the LF condition, division problems related to multiplication problems of the HF condition) as within-subject factor and group (younger adults, older adults) as between-subjects factor was performed on the arcsine-transformed mean proportion of correct answers. Results indicated a significant main effect of condition (Fig S2, panel a), *F*(2, 96) = 3.05, *MSE* = .40, *p* = .05, *µ_p_^2^* = .06. There was no significant main effect of group and no significant interaction between condition and group, *p* > .1. As indicated by pairwise comparisons with Bonferroni correction, there was a trend towards less accurate answers with division problems related to the LF multiplication condition than with unrelated division problems, *p* = .056. Unrelated division problems and division problems related to the HF multiplication condition were answered comparably accurately, *p* = 1. The difference between related conditions was also not significant, *p* = .294.

**RTs.** A mixed ANOVA with condition as within-subject factor and group as between-subjects factor on the ln-transformed mean RTs indicated a similar pattern of results. There was a significant main effect of condition (Fig S2, panel b), *F*(2, 94) = 4.86, *MSE* = .09, *p* = .01, *µ_p_^2^* = .09, whereas the main effect of group and the two-way interaction were not significant, *p* > .1. Pairwise comparisons with Bonferroni correction showed that division problems related to the LF multiplication condition were answered more slowly than division problems related to the HF multiplication condition, *p* < .05. Other contrasts were not significant, both *p* > .1.

**Fig S2. Mean percentage of correct answers (panel a) and mean reaction times in correct trials (panel b) as a function of condition (unrelated division problems, division problems related to the LF multiplication condition, division problems related to the HF multiplication condition). Bars indicate the standard error of the mean.**

**
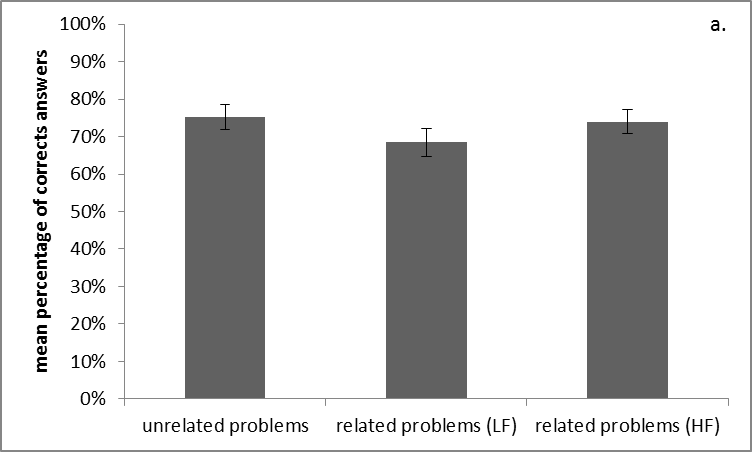

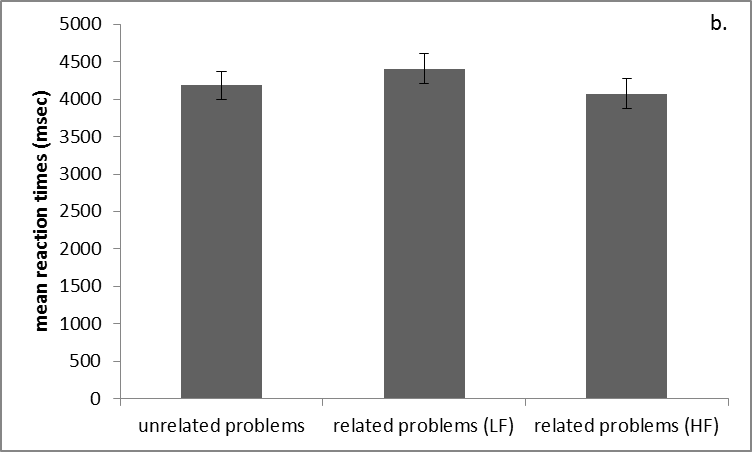
**
