# Supplementary material for: Arithmetic learning in advanced age
Source: PLoS One. 2018 Feb 28;13(2):e0193529. doi: 10.1371/journal.pone.0193529 (PMC5831411; doi:10.1371/journal.pone.0193529)
Supplement: S3 Results — (DOCX) [file pone.0193529.s003.docx]

**S3 Supporting Information - Accuracy and reaction times with “trained” multiplication problems during the five sessions of training**

**Results**

**Accuracy.** A mixed ANOVA with training session (S1, S2, S3, S4, S5) and condition (HF, LF) as within-subject factors, and group (younger adults, older adults) as between-subjects factor was performed on the arcsine-transformed mean proportion of correct answers. There was a highly significant main effect of session, *F*(4, 192) = 20.96, *MSE* = 1.02, *p* < .001, *µ_p_^2^* = .30. Pairwise comparisons with Bonferroni correction indicated a significant accuracy improvement from S1 to S2, *p* < .01; other contrasts were not significant, all *p* > .1. The mean accuracy rate was 88.49% (*SD* = 10.30) in S1, 91.61% (*SD* = 8.50) in S2, 93.37% (*SD* = 6.84) in S3, 93.38% (*SD* = 6.91) in S4, and 94.77% (*SD* = 6.34) in S5. The main effect of condition was also highly significant, *F*(1, 48) = 112.21, *MSE* = 9.66, *p* < .001, *µ_p_^2^* = .70, with participants responding overall more accurately to HF problems (*M* = 96.03% correct, *SD* = 4.81) than to LF problems (*M* = 88.62% correct, *SD* = 9.59). The main effect of group was not significant, *p* > .1. However, the inspection of the significant interaction between session and group, *F*(4, 192) = 2.98, *MSE* = .14, *p* < .05, *µ_p_^2^* = .06, by means of post-hoc comparisons with Bonferroni correction indicated a significant group difference in S2, *p* < .05, with the older group obtaining a higher accuracy rate than the younger group (Fig 1, panel a, in the main text). In other sessions, group differences were not significant, all *p* > .1. We found no other significant interaction, all *p* > .05.

**RTs.** A mixed ANOVA was also performed on the ln-transformed mean RTs in correct trials. We found a highly significant main effect of session, *F*(4, 192) = 138.14, *MSE* = 3.04, *p* < .001, *µ_p_^2^* = .74. Pairwise comparisons with Bonferroni correction indicated highly significant speed improvements across sessions, all *p* < .001. The mean response time was 3021.51 ms (*SD* = 768.00) in S1, 2647.64 ms (*SD* = 717.76) in S2, 2305.53 ms (*SD* = 603.54) in S3, 2112.33 ms (*SD* = 559.82) in S4, and 1992.04 ms (*SD* = 551.74) in S5. The main effect of condition was also highly significant, *F*(1, 48) = 317.70, *MSE* = 11.94, *p* < .001, *µ_p_^2^* = .87, with participants responding faster to HF problems (*M* = 2059.66 ms, *SD* = 515.70) than to LF problems (*M* = 2771.96 ms, *SD* = 685.48). Pairwise comparisons with Bonferroni correction were carried out to inspect the highly significant interaction between session and condition, *F*(4, 192) = 19.45, *MSE* = .11, *p* < .001, *µ_p_^2^* = .29. We found significant speed improvements across sessions with both LF problems (S1: *M* = 3297.07 ms, *SD* = 803.20; S2: *M* = 3056.08 ms, *SD* = 840.78; S3: *M* = 2713.15 ms, *SD* = 740.80; S4: *M* = 2478.74 ms, *SD* = 690.12; S5: *M* = 2314.74 ms, *SD* = 682.53) and HF problems (S1: *M* = 2745.95 ms, *SD* = 787.48; S2: *M* = 2239.20 ms, *SD* = 655.18; S3: *M* = 1897.90 ms, *SD* = 529.29; S4: *M* = 1745.91 ms, *SD* = 476.38; S5: *M* = 1669.34 ms, *SD* = 457.82), all *p* < .05. The main effect of group was significant, *F*(1, 48) = 6.45, *MSE* = 3.66, *p* = .014, *µ_p_^2^* = .12, such that the younger participants gave overall faster responses than the older participants (younger group: *M* = 2249.46 ms, *SD* = 604.82; older group: *M* = 2582.15 ms, *SD* = 523.69). We inspected the highly significant interaction between session and group, *F*(4, 192) = 7.66, *MSE* = .17, *p* < .001, *µ_p_^2^* = .14, by means of post-hoc comparisons with Bonferroni correction. Groups performed comparably fast during S1 and S2, both *p* > .05, while the younger group outperformed the older group in S3, S4, and S5, all *p* < .01 (Fig 1, panel b, in the main text). Other interactions were not significant, both *p* > .05.
